# Effectiveness of a New 3D-Printed Dynamic Hand–Wrist Splint on Hand Motor Function and Spasticity in Chronic Stroke Patients

**DOI:** 10.3390/jcm10194549

**Published:** 2021-09-30

**Authors:** Yu-Sheng Yang, Chi-Hsiang Tseng, Wei-Chien Fang, Ia-Wen Han, Shyh-Chour Huang

**Affiliations:** 1 Department of Occupational Therapy, College of Health Science, Kaohsiung Medical University, Kaohsiung 80708, Taiwan; yusheng@kmu.edu.tw; 2 Mechanical Engineering Department, National Kaohsiung University of Science and Technology, Kaohsiung 807618, Taiwan; jason012125@gmail.com; 3 Department of Physical Medicine and Rehabilitation, Kaohsiung Municipal United Hospital, Kaohsiung 80457, Taiwan; takako0703118@yahoo.com.tw; 4 Department of Physical Medicine and Rehabilitation, Kaohsiung Medical University Hospital, Kaohsiung 80756, Taiwan; yatwen1114@gmail.com

**Keywords:** stroke, spasticity, assistive technology

## Abstract

Spasticity, a common stroke complication, can result in impairments and limitations in the performance of activities and participation. In this study, we investigated the effectiveness of a new dynamic splint on wrist and finger flexor muscle spasticity in chronic stroke survivors, using a randomized controlled trial. Thirty chronic stroke survivors were recruited and randomly allocated to either an experimental or control group; 25 completed the 6-week intervention program. The participants in the experimental group were asked to wear the dynamic splint at least 6 h/day at home, for the entire intervention. The participants in the control group did not wear any splint. All the participants were evaluated 1 week before, immediately, and after 3 and 6 weeks of splint use, with the modified Ashworth scale and the Fugl−Meyer assessment for upper extremity. User experience was evaluated by a self-reported questionnaire after the 6-week intervention. The timed within-group assessments showed a significant reduction in spasticity and improvements in functional movements in the experimental group. We found differences, in favor of the experimental group, between the groups after the intervention. The splint users indicated a very good satisfaction rating for muscle tone reduction, comfort, and ease of use. Therefore, this new splint can be used for at-home rehabilitation in chronic stroke patients with hemiparesis.

## 1. Introduction

Post-stroke spasticity is a common complication associated with other signs and symptoms of upper motor neuron syndrome, including agonist and antagonist co-contraction, weakness, and lack of coordination. Stroke survivors with more severe paresis in the upper-limb muscles have a higher risk for developing spasticity in the arm, and contractures of the wrist and finger flexor muscles [1,2]. These problems often develop 6–8 weeks after a stroke [3,4].

Spasticity limits muscle lengthening, which can lead to two consequences. First, spastic muscles have a tendency to stay in a shortened position for longer periods, and second, voluntary activities of antagonist muscles are frequently restricted [5]. An implicit assumption exists that states that spasticity results in muscle fibers and connective tissue changes that can lead to contractures [6,7]. Spasticity and contractures in the upperlimb can significantly affect many activities of daily living or sleep, or can lead to a lesser ability to function [8,9,10]. Without appropriate attention, stroke survivors with spasticity are at risk for developing a clenched fist, a hand that is deformed into a fist by permanent shortening of the flexor muscles of the fingers and soft tissue [11].

When left in the immobilized state with the flexor synergy pattern after stroke, the condition of the upper limbs progresses to a fibrotic state, which triggers changes in the muscle fiber and sarcomere properties, and the development of early contractures [12]. In such conditions, prolonged muscle stretching is one of the most used treatments to manage spasticity and improve the viscoelastic properties of the muscle–tendon units [13,14]. Static stretching is a widely used type of prolonged muscle stretching, and may be applied in different ways, including by self-stretching, clinical therapist’s hands, splints, orthoses, or other physical modalities [13,14,15,16,17,18].

Splints can provide a safe low-load force to the spastic muscles, which facilitates muscle relaxation, maintains muscle length, and prevents contractures [19], thereby having widespread uses in many clinic settings. However, some previous studies did not support the effectiveness of hand splinting [20,21]. Systematic review research, conducted by Lannin and Herbert, concluded that there was insufficient evidence to either support or refute the effectiveness of hand splinting for adults, following stroke [22]. The effects of splinting may not be long lasting because of a failure to comply with the recommended procedures, and a lack of understanding of the effective dose of stretching within the rehabilitation process. A clear definition of “prolonged”, with regard to the duration of stretch, is not yet clear, and further research is required to determine the most appropriate technique and duration to produce the desired effect. Moreover, some studies were conducted with a static splint, an immobilization or supportive splint [20,21]. It has no moving components, and only provides support and immobilization. The position of the static splint sets the wrist/hand in a fixed position. However, the level of spasticity varies during daytime, resulting in different positions of the wrist/hand. The chosen position of the static splint might not be adequate to manage these varying levels of spasticity. By contrast, compared with static splints, a dynamic splint has a static base onto which levels, springs, or pulleys can be attached. It can deliver various stretching options, such as a prolonged or short duration with a high or low intensity of force to counteract the different levels of spasticity. As a result, a dynamic splint is superior, offering more benefits, such as reducing spasticity, allowing comfortable stretch, repositioning fingers in extension positions, and increasing hand performance [23].

In many of the cases we have observed, patients did not like to wear such splints or orthoses. About 33–50% of stroke patients did not wear static splints or orthoses daily for >8 has advised because of discomfort [24]. They complained about an increase in pain and spasticity, which makes it difficult to endure the splints or orthoses for a longer period each day [24]. This discomfort can be a result of the static characteristics of the splints. During moments with high levels of spasticity, the wrist tries to flex against the splints, which can cause pain, pressure sores over the bony prominences (e.g., radial or ulna styloid process), and even wrist or finger flexor hypertonicity, as a result of the pain stimulus. However, without an appropriate splint design to keep the wrist and fingers firmly in the stretched state, both will try to shorten, leading to a lack of stretch.

In recent years, the introduction of 3D-printing techniques in orthopedic and rehabilitation practices has been extensively discussed, because the use of such techniques renders it possible to customize splints or orthoses as well as enhance patient treatment satisfaction levels [25,26]. Varieties of 3D-printed splints have been reported to address deficiencies of the post-stroke spastic hand [16,27,28,29]. Most of them employed finger caps with elastic cords to stretch the fingers apart in an extension. However, when the degree of finger flexor spasticity is large, it becomes difficult to wear the splint with finger caps against the tension in stretching elastic cords simultaneously. Moreover, these 3D-printed splints had finger enclosures that did not allow the user to have any proprioceptive feedback when picking up the objects. Accordingly, proprioceptive feedback is essential for planning and controlling the limb postures and movements needed for the successful accomplishment of most common motor tasks [30,31]. Finger enclosures may hinder paretic limb detection ability, potentially leading to decreased finger movements.

To overcome the problems, we developed a dynamic splint based on a pulley rotation design. The results demonstrated the beneficial effects of this dynamic splint in eight chronic stroke patients, during a 4-week intervention [32]. However, the dynamic splint was found to have some limitations in some chronic stroke survivors. First, it was difficult to maintain the fingers firmly in the stretched state because the string tended to lose its tension when encountering cases of severe spasticity. In addition, chronic hemiparetic stroke patients were required to put each string of the finger cap on each finger, respectively, so that each finger could be stretched; they found it somehow difficult to handle these strings alone. To resolve these issues, we improved the design and simplified its operations. As a result, we developed a new dynamic splint using a four-bar linkage mechanism as a hand joint exoskeleton to fit the range of finger trajectories. This dynamic splint was intended to be effective in reducing spasticity, easy to fabricate, convenient to operate, and comfortable to wear. Moreover, based on our previous research, we observed that chronic stroke patients who wore the dynamic splints for longer than scheduled showed tremendous improvement, as compared to the group who had a schedule of wearing the splints 3 h a day [32]. A previous study had also indicated that the use of a dynamic splint for at least 6 h per day significantly reduced wrist contractures, causing less pain [17]. Therefore, the primary aim of this study was to evaluate the effect of this new dynamic splint on hand motor function and spasticity in chronic hemiparetic stroke survivors. The secondary aim was to describe the self-reported experiences after the use of this new dynamic splint. We hypothesized that these stroke survivors would be able to relieve their wrist and finger flexors hypertonia, thereby enhancing hand motor function after wearing this dynamic splint. They were able to endure this splint for the prescribed 6 h a day without discomfort.

## 2. Materials and Methods

### 2.1. Participants

Thirty participants were recruited from outpatients visiting in the Kaohsiung Medical University Hospital. Participants were included if they met the following criteria: (1) were >18 years of age, (2) had a first-ever stroke resulting in upper-limb spastic hemiplegia >1year before admission to the study, and (3) had upper-limb spasticity (modified Ashworth scale (MAS) scores of 1–3 at the wrist and/or finger flexors). Participants were excluded if they (1) had deficits in language or cognitive impairments that were likely to interfere with their cooperation in the study, (2) presented with severe upper-limb contractures, and (3) had received botulinum toxin injections <6 months prior to study admission. Participants taking oral anti-spastic drugs were only included in the study if the dosage had not been changed during the month before joining. All participants provided informed written consent. The study was conducted in accordance with the Declaration of Helsinki Ethical Principles and Good Clinical Practices and was approved by the Institutional Review Board of Kaohsiung Medical University Chung-Ho Memorial Hospital (KMHIRB-F(I)-20200017).

### 2.2. Intervention

We randomly assigned participants following a simple randomization procedure (computerized random numbers) to an experimental or a control group. Both groups received the conventional rehabilitation therapy by experienced therapists, including putting patient’s limbs in a normal position, the stretching technique of the wrist/finger flexor muscles, proprioceptive neuromuscular facilitation (PNF), neurodevelopmental technique (NDT), and task-oriented training to enhance the motor function and sensory function of the wrist and hand, and activities of daily living training for upper limb. Conventional rehabilitation therapy was performed 3 times per week, for 40 min per time during the 6-week intervention. Participants in the experimental group were asked to wear a custom-made, dynamic 3D-printed hand–wrist splint for at least 6 h per day at home for the 6-week intervention. Participants or caregivers who helped apply the splint self-recorded their actual daily splint wearing time in a diary. Participants in the control group did not wear a hand splint for the study period and were advised to stretch their wrist or finger flexors in a home exercise program.

**Figure 1:**
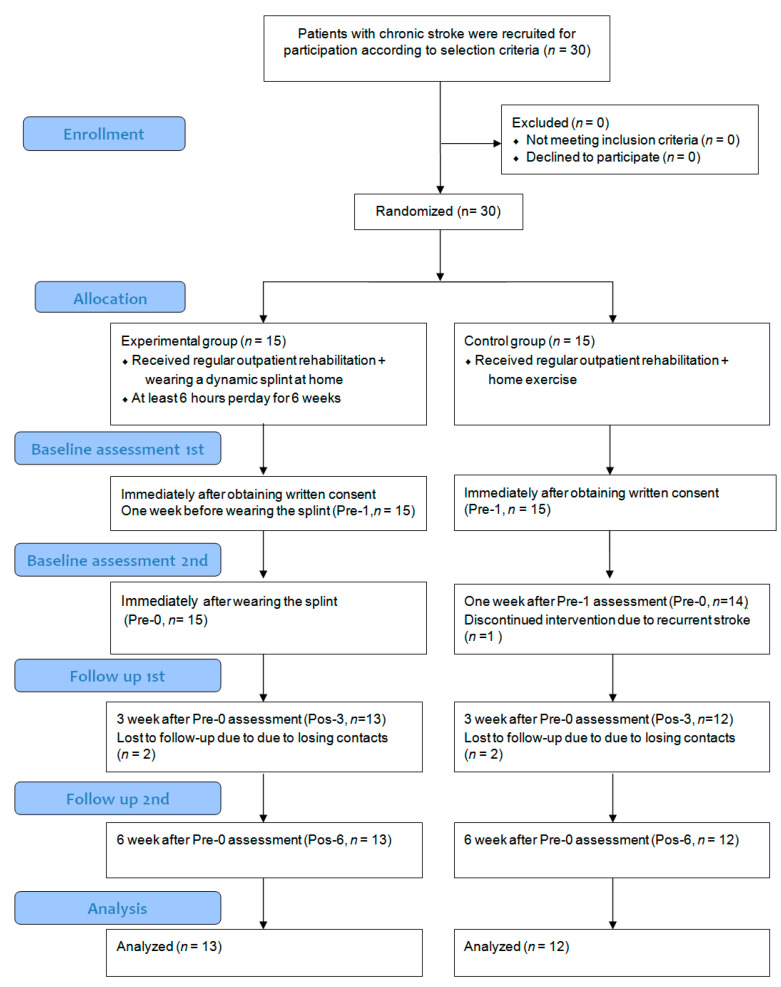
Experiment flowchart.

The study flowchart is illustrated in Figure 1. We assessed the participants in both groups four times. All participants were assessed twice before wearing the dynamic splint within an interval of 1 week and assessed within a 3-week interval for 6 weeks after wearing the splint (first assessment: Pre-1; second assessment: Pre-0; third assessment: Pos-3; fourth assessment: Pos-6). The multiple-baseline design (Pre-1, Pre-0) used in this study enabled us to map improvements on the basis of changes in outcome measures.

### 2.3. D-Printed Dynamic Splint

**Figure 2:**
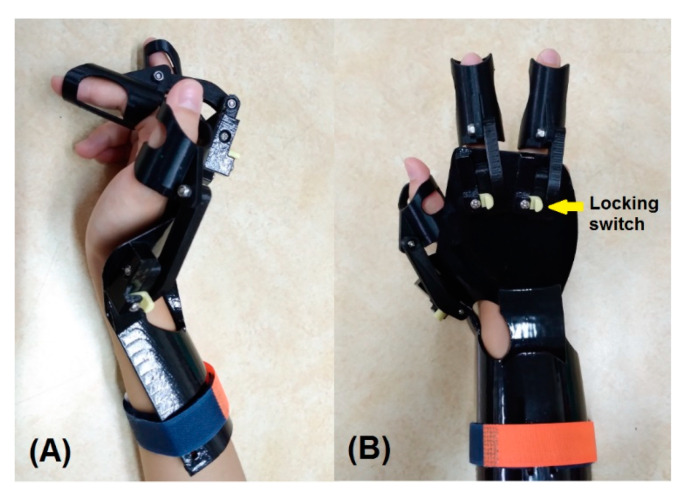
(**A**) Lateral view and (**B**) dorsal view of a 3D-printed dynamic hand–wrist splint.

The 3D-printed dynamic splint consisted of a modified dorsal wrist splint, link bars, and finger caps (Figure 2). This splint held the wrist extended to a 45-degree position. To address the many practical and anatomical challenges of the hand exoskeleton design, we used a four-bar linkage mechanism as a hand joint exoskeleton to fit the range of finger trajectories [33]. The linkage was located at the dorsal wrist and attached to the medial phalanx of the finger just above the distal interphalangeal joint with a finger cap. We designed the mechanism so that it did not interfere with the finger motion, and the user’s fingertips and palm were free to touch real objects and experience tactile feedback. In addition, the fingers could be stretched in extension with the wrist, extended by a simple locking mechanism for a prolonged time. Our previous study had shown that stretching three fingers (thumb, index, and middle fingers) reduced the finger flexors’ spasticity, and the splint was easy to self-use at home [32]. Therefore, we used a 3D-printed dynamic splint with a novel three-finger design in the current study.

This study used a fused deposition modeling (FDM)-based 3D printer (UP Box, Go Hot Technologies Co., Ltd., Taiwan) to print all components of this dynamic splint. Each individual part was printed with acrylonitrile–butadiene–styrene (ABS) filament, which has excellent mechanical strength and durability properties. The printing parameters were set as follows: layer thickness of 0.2 mm, 20% filling level, printing temperature of 230 °C, and printing speed of 60 mm/s. Our dynamic splint was customized and manufactured solely on the basis of participant’s anthropometric measurements, such as the width of forearm, wrist and fingers; the length of middle finger, index, and thumb. The customized dynamic splint can be manufactured in only 10 h once all the parameters of the participants are measured. The total estimated cost of this dynamic splint is roughly USD 80. This includes the price of all components needed to fabricate the splints, with the exception of the 3D printer. However, considering that the amount of filament required for each splint is estimated to be between 100 and 150 g, depending on its size, the estimated price is expected to decrease further.

### 2.4. Outcome Measures

The primary outcome measure was the MAS for evaluation of the severity of wrist and finger flexor spasticity. The MAS, a six-category ordinal scale used to assess the resistance encountered during a passive muscle quickly stretching, not requiring instrumentation [34], is the most commonly used tool for evaluating the efficacy of pharmacological and rehabilitation interventions for spasticity among patients with stroke. To allow for the analysis by the statistical software, we modified the MAS scores 1+ to 4, to 2–5 for our analysis [15,18,35]. A smaller score indicated better improvement of spasticity release. Inter- and intra-rater agreement for the MAS measuring upper extremities was 0.78 and 0.84, respectively [36].

Another primary outcome measure was the Fugl–Meyer assessment for upper extremity (FMA-UE), which is the “gold standard” for assessing the motor recovery of post-stroke hemiparesis in clinical trials [37,38]. The FMA-UE consists of 30 items assessing motor function and 3 items assessing reflex function with total scores ranging between 0 and 66. Higher FMA-UE scores mean better motor function. Inter- and intra-rater agreement for the FMA-UE was 0.95 and 0.98, respectively [39,40,41].

A secondary outcome measure was a subjective self-reported questionnaire regarding pain, spasticity, satisfaction, and ease of self-wear. We measured each item as well with a 10cm visual analog scale(VAS), with 0 cm representing “no pain”, “no spasticity”, “extremely dissatisfied”, or “extremely hard” and 10 cm representing “worst pain”, “worst spasticity”, “very satisfied”, or “very easy”. A senior therapist in stroke rehabilitation who was blinded to the group identities conducted all the outcome measures.

### 2.5. Statistical Analysis

The sample size was calculated based on G power software version 3.1.1 with expected differences between experimental and control groups on the MAS scale from a previous study [15]. The sample size calculation indicated that 15 patients per group should have been enrolled for α = 0.05 and 1–β = 0.95, with the effect size of 1.282. Therefore, the indicated overall number of 30 participants was achieved in this study. To summarize the results of the duration of wearing time, pain, subjective spasticity, level of satisfaction, and the easy self-wear descriptive statistics were used. Quantitative variables were compared using independent t-test/Mann–Whitney U test (when the data sets were not normally distributed) between the two groups. Categorical variables were compared using chi-square test. Analysis of covariance (ANCOVA) was used to compare MAS and FMA-UE scores at multiple time points between two groups after adjusting for the baseline values. Repeated measures analysis of variance (ANOVA) with Bonferroni adjustment was used for comparison of MAS and FMA–UE scores within each group between multiple time points. All statistical analyses were performed using the SPSS software (version 20 for Windows, IBM, Armonk, NY, USA), and the level of significance was set to 0.05.

## 3. Results

### 3.1. Participant Characteristics

Thirty stroke survivors were recruited in this study, but only 25 completed the 6-week intervention. One participant in the control group dropped out because of recurrent stroke, and four participants (three in the experimental group, one in the control group) were lost during follow-up. The participants’ demographic and clinical characteristics are presented in Table 1. No significant demographic differences occurred between the groups, including age, gender, stroke history, and affected side.

### 3.2. Clinical Assessments

**Figure 3:**
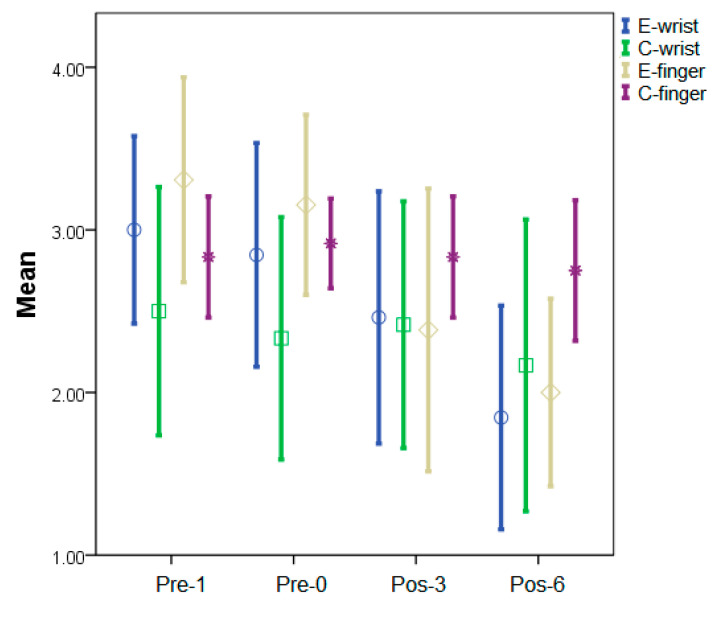
Sequential changes in mean MAS scores for the wrist and finger flexors in the study. Mean values and ±1 standard error were displayed. E-wrist: wrist flexors in the experimental group; C-wrist: wrist flexors in the control group; E-finger: finger flexors in the experimental group; C-finger: finger flexors in the control group.

The average MAS scores of the wrist and finger flexors at different assessments in the study groups are shown in Table 2. Within the experimental group, we found no significant differences in the wrist and finger flexors between Pre-1 and Pre-0. However, there was a significant decrease in the finger flexors at Pos-3 (*p* = 0.03) and at Pos-6 (*p* < 0.01 on wrist flexors, *p* < 0.01 on finger flexors), respectively, when compared to Pre-0. Within the control group, we found no significant serial MAS changes in either the wrist or finger flexors at Pre-1, Pre-2, Pos-3, and Pos-6, although we observed a slow decreasing tendency (Figure 3). Moreover, after 3 weeks of the wearing intervention, the average MAS of the finger flexors showed a significant decrease (*p* = 0.05); after 6 weeks, the average MAS of the wrist (*p* < 0.01) and finger (*p* < 0.01) flexors in the experimental group were significantly decreased compared with those in the control group.

**Figure 4:**
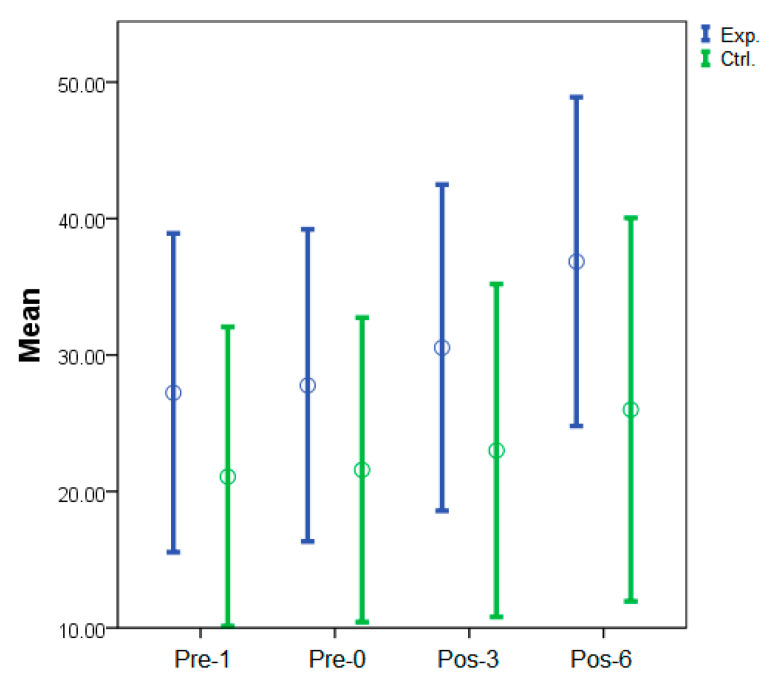
Sequential changes in mean FMA-UE scores in the study groups. Mean values and ±1 standard error were displayed. Exp.: experimental group; Ctrl.: control group.

The average FMA-UE scores at different assessments in the study groups are shown in Table 3. Similarly, for the experimental group, the average FMA–UE scoreat Pre-1 and Pre-0 showed no significant differences. We observed a significant improvement in Pos-3 (*p* = 0.02) and Pos-6 (*p* < 0.01), when compared to Pre-0. For the control group, we found no significant differences in the FMA-UE scores between Pre-1 and Pre-0. However, we noted a slight improvement in Pos-3 (Figure 4). Thereafter, we found a significant increase until Pos-6 (*p =* 0.01). Moreover, we found no significant differences in the FMA-UE scores in the experimental group, when compared to the control group, at Pre-1, Pre-0, and Pos-3. We did find a significant difference until Pos-6 (*p =* 0.05).

### 3.3. Subjective Self-Reported Findings

The participants in the experimental group, at 6 weeks, showed significant differences in their attitudes towards splint wearing time (*p* < 0.01), reduced spasticity (*p* < 0.01), level of satisfaction (*p* < 0.01), and ease of use (*p* < 0.01) (Table 4). However, the pain ratings showed no statistical differences between Pos-3 and Pos-6 (*p* = 0.89). The participants reported that they had still experienced mild, annoying pain after wearing the splint for 6 weeks.

## 4. Discussion

The results from this study indicated that our new 3D-printed dynamic hand–wrist splint was effective in significantly reducing wrist and finger flexor spasticity after 3 weeks of intervention. Furthermore, we observed a further effect on reducing spasticity after wearing the splint for 6 weeks. We also found significant alleviation in self-reported spasticity after 6 weeks of intervention.

Several previous studies have described a variety of dynamic splints that effectively improved motor function and relieved spasticity in stroke patients [16,17,32,42]. In comparison to a static splint, a dynamic splint consists of a static base that forms the foundation of the splint, and an outrigger, the mobile part consisting of levers, springs, or pulleys. It also included a dynamic component to facilitate splint mobility with the associated structures, such as finger springs and caps, an adjustable tensioner, and wrist mount areas. This dynamic component offers energy-storing properties and provides various resistance grades against spasticity. Therefore, as flexor tone increases in the patient’s hand, the fingers remain within the splint instead of pulling out. The dynamic hand piece then repositions the fingers into extension, as tone increases.

Thus, a dynamic splint is far superior to a static splint because it provides more benefits, such as reducing spasticity, allowing for comfortable stretch, and repositioning fingers into extension positions. Many dynamic splints for reducing hand spasticity have been developed; however, most of these were too complicated to wear, involving a glove or hook-and-loop fasteners strapping each finger in firmly. Often, it is technically difficult for a stroke patient with spasticity to wear these types of splints alone at home.

The new dynamic hand–wrist splint presented in this study has a simple device configuration and low-cost fabrication. The finger cap has a similar design to the finger sleeve, for easy and quick wearing or removing. Furthermore, this splint uses a four-bar linkage mechanism to fit finger trajectories and caps to reposition the fingers. Therefore, it allows for a comfortable prolonged stretch and maintains the fingers in an extended position to decrease wrist and finger flexor spasticity.

The motor function of the affected wrist and hand (measured with the FMA-UE) showed statistically significant improvements after 3 weeks of intervention and had sustained improvement at 6 weeks. The average difference in improvement between Pre-0 and Pos-6, using the FMA-UE, was 9.1, which we believed to be clinically meaningful because the minimal clinically important difference of the FMA-UE has been established to be between 4.25 and 7.25 for patients with chronic stroke [43]. Moreover, 76.9% of the participants in the experimental group exceeded the MCID of 4.25. The increase in the FMA-UE score demonstrated the voluntary motor improvements achieved by the participants.

Historically, traditional rehabilitation approaches have focused on reducing spasticity as a prerequisite for improving motor function [44]. In stroke survivors with functionally useful voluntary limb movement, inappropriate co-activation of agonist and antagonist muscles can cause spastic co-contraction, thereby impeding normal limb movement [45,46]. One possible reason for the better performance in the FMA-UE score with decreased MAS in the experimental group was that the participants could be able to exert more voluntary effort in the reach-to-grasp tasks. People with stroke often have difficulty generating force in the finger extensor to open their hands, due to stronger wrist and finger flexor spasticity. The suppression of wrist and finger flexor spasticity, by prolonged stretching with a dynamic hand–wrist splint, can decrease the ability to counteract the agonist wrist and finger extensor muscles, thereby increasing the ability to voluntarily control hand opening. Furthermore, maximized involvement of voluntary effort in post-stroke limb practice has been found to be an important factor related to the significant release of muscle tone, with long-term effects [47]. When the affected hand was able to start performing the tasks they could not achieve (e.g., hand open) by themselves, they would be promoted to practice reach-to-grasp tasks. Our findings are also consistent with other robot-assisted therapy in upper extremity hemiparesis studies, suggesting that decreased muscle tone occurs in chronic stroke individuals with better voluntary motor functions [47,48,49].

Both groups received approximately 2 h of upper-limb therapies per week, as conventional stroke rehabilitation programs. The conventional program is patient-specific, task-specific, and consists of PNF and NDT. Presumably, those conventional treatments had a relevant influence on the patients’ improvement. Consequently, the FMA–UE scores of the control group gradually increased by 4.5 points, from the baseline to Pos-6, but these scores were not superior to those in the experimental group. The use of a 3D-printed dynamic hand–wrist splint at home, combined with a conventional rehabilitation program, could provide additional long-term benefits in terms of upper extremity motor recovery and motor functioning.

The participants in this study reported a significant difference in reducing spasticity and in increasing wearing time before and since the use of this splint. The participants were willing to wear the splint from 6 h, as advised, to 7.9 h/day after the 6 weeks of intervention. The participants reported high levels of satisfaction and ease of use, finding it easy to wear this dynamic splint on their affected hand independently, without any problems.

The self-reported pain did not show a significant difference, although the spasticity complaints tended to decrease. Therefore, our new dynamic splint, using prolonged stretching principles to guard against increased spasticity, muscle stiffness, and shortening in the wrist and finger flexors, certainly caused some pain. Yet, after wearing the splint for 6 weeks, not one of the participants ceased wearing it because of a painful experience. On the contrary, the participants wore this splint for longer than the advised hours. One possible reason for this might be that these stroke patients experienced improvement in their hand function and thus were willing to keep wearing the splint.

The strength of our study is that it used a randomized controlled trial design. We selected the FMA–UE and MAS to measure primary outcomes and a self-reported questionnaire to measure secondary outcomes. However, there were limitations that need to be addressed in this study. First, this study was limited in that we surveyed the experimental group using a self-reported questionnaire, which is not a standardized questionnaire. This survey may simply lack sufficient validity and reliability. However, the self-reported data collected by this survey used VAS, which is a simple and frequently used method to evaluate variations in satisfaction or discomfort rating questionnaires. These self-reported outcomes may still give useful insights into the user’s perspective on this dynamic splint. Another limitation was that the included participants were stroke patients; they often have a certain degree of cognitive impairment. We did not assess their cognitive abilities for both groups; however, we ensured that the participants in the experimental group understood how to wear this dynamic splint properly.

One more limitation was the choice of MAS as the spasticity assessment scale. The MAS is a muscle tone assessment scale used to assess the resistance experienced during a passive range of motion. It is the most commonly used tool to evaluate the efficacy of pharmacologic and rehabilitation interventions for the treatment; therefore, it can be easily compared with the results obtained in other studies. However, recent studies raised questions about the moderate inter- and intra-rater reliability [50,51] and validity of the MAS assessment of spasticity [51,52]. Spasticity, defined as hyper-resistance measured during passive rotation of a joint, is related to neural and non-neural factors. MAS does not address the velocity-dependent aspect of spasticity [51,52], and it has been described as a grading of muscle stiffness to solely assess resistance to passive movement [51]. Despite its popularity, MAS has been subject to criticism; the modified Tardieu scale (MTS) has been suggested as an alternative and suitable measure for use in the assessment of spasticity [52,53]. MTS could address the intensity of the resistance, first-noticed catch angle, clonus, and differences among joints and muscles that move at different velocities [52,53]. Nevertheless, MAS generally reflects muscle overactivity, including the elements of hypertonia and muscle stiffness, which are both the components of a positive sign of upper motor neuron syndrome, and they compose the resistance of passive stretch. These elements affecting the active or passive function of patients could be treated through pharmacologic treatment or other remedies. Therefore, it is reasonable to use the MAS as a scale for measuring muscle tone [54].

Finally, our results are limited to stroke survivors with mild–moderate upper-limb impairments. This dynamic splint can only be applied to stroke survivors who can extend their wrist and open their affected hand by passive movements. Therefore, the results cannot be generalized to other stroke populations, especially those with severe spasticity of the affected limb.

## 5. Conclusions

In conclusion, this new 3D-printed dynamic hand–wrist splint is a feasible and effective alternative modality for reducing muscle spasticity and improving hand motor function. The users gave very good satisfaction scores for muscle tone reduction, comfort, and ease of use. This splint can be a supplementary device for home exercises in addition to hospital-based rehabilitation for chronic stroke survivors with hemiparesis.

## Figures and Tables

**Table 1:** Participant demographic characteristics and clinical features between the groups.

Characteristic/Feature	Group		
	Experimental (*n* =13)	Control (*n* =12)	*p*-Value
Mean age, years	44.4 (10.2)	47.1 (9.2)	0.55
Gender (male/female)	11/2	10/2	0.93
History of stroke, months	22.5 (11.9)	20.0 (10.4)	0.48
Affected side (right/left)	7/6	6/6	0.86

Values are presented as means (standard deviations).

**Table 2:** Average MAS scores of the wrist and finger flexors in the study groups.

Group	Pre-1	Pre-0	*p*-Value ^a^	Pos-3	*p*-Value ^a^	Pos-6	*p*-Value ^a^
E-wrist	3.0 (0.6)	2.8 (0.7)	0.99	2.4 (0.8)	0.57	1.8 (0.7)	<0.01 *
C-wrist	2.5 (0.8)	2.3 (0.8)	0.99	2.4 (0.8)	0.9	2.2 (0.9)	0.99
*p*-value ^b^	0.92	0.92	-	0.57	-	<0.01 *	-
Partial EtaSquared	<0.01	<0.01		0.02		0.28	
E-finger	3.3 (0.6)	3.2 (0.6)	0.99	2.4 (0.9)	0.03 *	2.0 (0.6)	<0.01 *
C-finger	2.8 (0.4)	2.9 (0.3)	0.99	2.8 (0.4)	0.99	2.8 (0.5)	0.99
*p*-value ^b^	0.22	0.22	-	0.05 *	-	<0.01 *	-
Partial Eta Squared	0.07	0.07		0.17		0.61	

Values are presented as means (standard deviations); * *p* < 0.05. E-wrist: wrist flexors in the experimental group; C-wrist: wrist flexors in the control group; E-finger: finger flexors in the experimental group; C-finger: finger flexors in the control group. ^a^ *p*-value was determined by ANOVA using Bonferroni adjustment; ^b^ *p*-value after adjusting for multiple baseline using ANCOVA between experimental group and control group at each assessment.

**Table 3:** Average FMA-UE scores in the study groups.

Group	Pre-1	Pre-0	*p*-value ^a^	Pos-3	*p*-Value ^a^	Pos-6	*p*-Value ^a^
Exp	27.2 (11.4)	27.8 (11.7)	0.07	30.5 (12.0)	0.02 *	36.8 (11.2)	<0.01 *
Ctrl	21.1 (11.6)	21.6 (11.5)	0.13	23.0 (12.7)	0.06	26.0 (14.7)	0.01 *
*p*-value ^b^	0.89	0.89		0.39	-	0.05 *	-
Partial EtaSquared	<0.01	<0.01		0.03		0.17	

Values are presented as means (standard deviations); * *p* < 0.05. ^a^ *p*-value was determined by ANOVA using Bonferroni adjustment; ^b^ *p*-value after adjusting for multiple baseline using ANCOVA between experimental group and control group at each assessment.

**Table 4:** Subjective reported wearing time, pain, spasticity, level of satisfaction, and ease of self-wear in the experimental group.

Time (*n* =13)	Wearing Time (h)	Pain (0–10)	Spasticity (0–10)	Satisfaction (0–10)	Easy to Use (0–10)
Pos-3	7.2	2.6	7.4	7.7	8.0
Pos-6	7.9	2.5	5.6	8.4	8.8
*p*-value	<0.01 *	0.89	<0.01 *	<0.01 *	<0.01 *

Values are presented as means; * *p* < 0.05.

## Data Availability

Data available on request due to privacy/ethical restrictions. The data presented in this study are available on request from the corresponding author.
